# Association of the rs555172 polymorphism in SENCR long non-coding RNA and atherosclerotic coronary artery disease

**DOI:** 10.15171/jcvtr.2017.29

**Published:** 2017-09-30

**Authors:** Nahid Shahmoradi, Mahboobeh Nasiri, Hajar Kamfiroozi, Mohammad Ali Kheiry

**Affiliations:** ^1^Department of Biology, Islamic Azad University, Arsanjan Branch, Arsanjan, Iran; ^2^Department of Cardiology, Shiraz University of Medical Sciences, Shiraz, Iran; ^3^Internist-Cardiologist, Private Dr. Kheiry Clinic, Neyriz, Fars, Iran

**Keywords:** Coronary Artery Disease, Polymorphism, SENCR, lncRNA

## Abstract

***Introduction:*** Variants in long non-coding RNAs (lncRNAs) have been implicated as potential biomarkers in prediction of complex disorders such as coronary artery disease (CAD). Studies considering the impact of the SENCR antisense lncRNAs on CAD have not established yet in Iranian population. This study aimed to investigate the association between SENCR rs555172 polymorphism and CAD in south Iranian population.

***Methods:*** Amplification refractory mutation system-polymerase chain reaction (ARMS-PCR) was performed to determine the allele and the genotype distribution of SENCR lncRNA polymorphism in 150 patients with CAD compared with 149 healthy controls through this hospital-based case-control study.

***Results:*** The frequency of AA, AG, and GG genotypes in cases were 32.7%, 44.7%, and 22.6%, and in controls were 26.8%, 49%, and 24.2%, respectively. Association was not found with any of the genotypes in comparison of cases and controls. The allelic frequencies did not differ between cases and controls. Cross-tabulating the population based on the gender, the frequency of the GG genotype was significantly higher among women of the case group compared to men. The difference was not seen in the control group between two sexes.

***Conclusion:*** The results suggested that the SENCR gene polymorphism did not confer susceptibility to CAD.

## Introduction

Coronary arteries are responsible for bringing oxygen-rich blood to the heart. Hardening of the coronary artery or “atherosclerosis” is a major cause of disturbance in heart muscles feeding and cardiovascular disorders like coronary artery disease (CAD).^1^ This complex and ordered process, including immunological inflammation, lipid oxidation and proliferation of smooth-muscle cells (SMCs) leads to marked narrowing of the coronary arteries.^2,3^ Hypercholesterolemia, hypertension, diabetes mellitus, and cigarette smoking are the best-known modifiable risk factors for CAD.^4^ Despite several studies on the identification of CAD-related genes and their polymorphisms,^5^ researches investigating the role of long non-coding RNA (lncRNA) polymorphisms on the susceptibility to CAD are very limit. lncRNAs are a heterogeneous group of transcripts and are the vital components of the gene regulatory network. They comprise a wide range of functions, including structural or trafficking roles, controlling cell cycle, differentiation, maintenance of the integrity of the cells and tissues and apoptosis.^6,7^ Although the influence of lncRNAs in heart development and cardiovascular disease is not completely clear, but the results of the most recent studies have discovered some clue in favor of their impact on the cardiovascular system.^8^

Smooth muscle and endothelial cell–enriched migration/differentiation-associated long non-coding RNA (SENCR; ID:100507392,) which is also named FLI1-AS1 or lncRNA9, is highly expressed in endothelial cells, SMCs and aortic tissue.^9^ The gene coding for this non-protein coding transcript is located on chromosome 11 (11q24.3) and containing three exons.^10^ SENCR is a non-natural antisense transcript oriented in the reverse direction of one of the FLI1 (friend leukemia virus integration 1) gene intron. SENCR implicated the cytoplasmic localization with no influence on the expression of the overlapping FLI1 gene.^9,11^ The best-characterized function of the SENCR is the maintenance of the contractile phenotype of the human smooth muscle cells, further, it can function as a decoy of miRNAs or a regulatory partner for several mRNAs or proteins.^11,12^

Afterwards, the current study intended assessing the association of SENCR rs555172 gene polymorphism with the risk of CAD in a sample of the Iranian population mostly from south of Iran.

## Materials and Methods

### Study subjects

Totally, 299 subjects including 150 patients diagnosed with CAD and 149 healthy subjects as a control group were investigated in this hospital-based case-control study from September 2015 to March 2016. Patients were diagnosed by an expert cardiologist among the ones hospitalized in angiography ward according to the presence of angiographically determined atherosclerotic lesions in coronary vessels. The inclusion criteria were; age less than 45 years for men, and 55 years for women, lack of the existence of any known risk factor for CAD including; the cigarette smoking, hypercholesterolemia, diabetes mellitus, hypertension and family history of CAD in first-degree relatives. Current smokers were considered as positive smoking status, and who never smoke as a negative group. Hypercholesterolemia was considered as either under treatment with lipid lowering drugs or having a total cholesterol greater than 200 mg/dL. Blood pressure greater than 140/90 mm Hg or even being on therapy considered as hypertension. Diabetes mellitus was diagnosed as a fasting blood glucose level >126 mg/dL or dependency to insulin or additional hypoglycemic drugs. If there was more than one patient in the family, only one recruited to the study and the others were excluded. The control subjects were selected from healthy blood donors based on not having the previous history of CAD or any other heart disorders, and further clinical situation like hypercholesterolemia, diabetes mellitus, and hypertension. They were all from the families with no affected members and matched on ethnicity, gender, age, and geographical area with the case group.

Demographic characteristics of the study population were collected through oral question and answer and recorded on the questionnaire. All subjects signed the written informed consent form. The procedure of the projects performed under the consideration of the research committee of Islamic Azad University, Arsanjan Branch.

### Molecular analyses

After extraction of the pure genomic DNA using salting out method, amplification of the genomic region containing rs555172 single nucleotide polymorphism (SNP) was done using the amplification refractory mutation system-polymerase chain reaction (ARMS-PCR). Two independent PCR reactions were done for each sample adding a pair of common primer and an allele-specific primer in each tube. The allele-specific primers just differed in the 3′-end nucleotide (A or G in the case of rs555172 polymorphism). The list of primers is as follows: forward common: 5′-GTG TCT CTG TAT TTC TGG GTT TAG-3′, reverse common: 5′-CAG TGT AGG GCT GGA TTT AGT G-3′, A-allele specific primer: 5′-TGA AGG TAG CTC TGT GGG TAC T**T**-3′, G-allele specific primer: 5′-GAA GGT AGC TCT GTG GGT ACT **C**-3′. Ready to use PCR master mix (Yekta Tajhiz Azma, Iran) was used following the manufacturer’s instruction. The optimal annealing temperature was 60°C. The PCR products were resolved on 2% agarose gel electrophoresis and amplicons detection was done using DNA safe stain dye.

### Statistical analyses

The goodness of fit between the observed number of each genotype and those expected theoretically was investigated using a chi-square test. Independent sample *t* test was used to compare the mean differences of age between cases and controls. The possible association between the polymorphism and CAD susceptibility was tested doing regression analysis calculating OR and 95% CI. The *P* value less than 0.05 considered significant. Data analyses using IBM SPSS statistics 19.

## Results

The baseline characteristics of the study subjects are shown in Table 1. The mean age of both cases and controls were approximately equivalent and did not show statistical differences (*P *= 0.69). The sex ratio (male to female) in case group was 105/45≈2.3.

**Table 1:** Demographic characteristics of the study population

**Characteristics**	**Controls**	**Cases**	***P***
Total number	149	150	-
Age (mean ± SD) (y)	51.94 ± 10.15	51.45 ± 10.70	0.69^a^
BMI (kg/m^2^)	26.40 ± 3.72	25.50 ± 3.50	0.03
Sex ratio (M/F)	99/50	105/45	-

Abbreviations‏: SD, standard deviation; F, female; M, male. ^a^Student *t* test.

Distribution of genotypes in both cases (χ^2^= 1.43, *df* = 1, *P *= 0.23) and controls (χ^2^= 0.05, *df* = 1, *P *= 0.81) were consistent with the expected ones based on Hardy-Weinberg equilibrium. Evaluating the influence of rs555172 genotypes on the risk of CAD using binary logistic regression resulted in no association between genotypes and alleles with the disease (Table 2).

**Table 2:** Comparison of the allele and genotype frequencies of the rs555172 polymorphism between cases and controls

**rs555172**	**Control** ** No. (%)**	**Cases** ** No. (%)**	P*	**OR**	**95% CI**
**All subjects**					
AA	40 (26.8)	49 (32.7)	-	1	Reference
AG	73 (49)	67 (44.7)	0.29	0.75	0.44- 1.30
GG	36 (24.2)	34 (22.6)	0.42	0.77	0.41- 1.4
AG+GG	109 (73.2)	101 (67.4)	0.27	0.76	0.46- 1.24
**Allele**					
A	153 (0.51)	165 (0.55)	-	1	Reference
G	145 (0.49)	135 (0.45)	0.37	0.86	0.63- 1.19

* Logistic regression analysis; *P* ≤ 0.05 considered significant.

In further investigation, the distribution of alleles and genotypes were calculated in patients and healthy subjects between two gender groups (men/women) separately. See the results in Table 3. The frequency of the genotypes in healthy subjects was not differed between men and women, while the frequency of the GG genotype was higher in women (33.3%) compared to men (18.1%) in patients. This difference showed statistical importance (OR: 3.07, 95%CI: 1.16-8.12, *P *= 0.02). This difference disappeared in the comparison of the allelic frequencies between males and females (OR: 1.84, 95%CI: 1.12-3.03, *P *= 0.07).

**Table 3:** Distribution of allele and genotypes of rs555172 polymorphism in cases and controls

**Groups**	**Male** ** No. (%)**	**Female** **No. (%)**	***P*** ^*^	**OR**	**95% CI**
***Controls***	*Genotypes*					
	AA	27 (27.3)	13 (26)	-	1	reference
	AG	52 (52.5)	21 (42)	0.67	0.84	0.36- 1.93
	GG	20 (20.2)	16 (32)	0.28	1.66	0.65- 4.22
	AG+GG	72 (72.7)	37 (74)	0.86	1.06	0.49- 2.31
	*Allele*					
	A	106 (0.54)	47 (0.47)	-	1	reference
	G	92 (0.46)	53 (0.53)	0.28	1.29	0.80- 2.10
***Cases***	*Genotypes*					
	AA	39 (37.1)	10 (22.2)	-	1	reference
	AG	47 (44.8)	20 (44.4)	0.25	1.66	0.69- 3.96
	GG	19 (18.1)	15 (33.3)	0.02	3.07	1.16- 8.12
	AG+GG	66 (62.9)	35 (77.7)	0.07	2.06	0.92- 4.63
	*Allele*					
	A	125 (0.60)	49 (0.44)	-	1	reference
	G	85 (0.40)	50 (0.56)	0.07	1.84	1.12- 3.03

* Logistic regression analysis; *P* ≤ 0.05 considered significant.

## Discussion

We attempted to investigate the association between polymorphism of SENCR antisense lncRNA and CAD. The frequency of genotypes among patients was the same as those calculated in controls and the observed difference was not statistically significant. In the independent comparison of the allele and genotype frequency among men and women in case and control groups, the frequency differences between two sexes were significant, and the GG genotype was more frequent among women suffer from CAD in comparison to the sick men.

The impaired function and structure of endothelial is linked with CAD and may contribute to the pathogenesis of the cardiovascular disorder.^13^ On the other hand, SMC contractile phenotype and under controlled proliferation are crucial in the prevention of atherosclerosis.^14^ SENCR is the mostly transcribed lncRNA in endothelial cells and responsible for maintaining proper contraction of smooth muscle cells was described for the first time in the year 2014.^9^ Although, the exact mechanism of action of the SENCR remains unclear, especially in endothelial cells, the results of knocking down experiments indicate interesting data about the function of the SENCR transcript in the cardiovascular system. SENCR involved in the regulation of migration and differentiation in SMCs and endothelial cells by controlling the expression of pre-migratory genes, e.g. PTN and MDK and contractile gene such as MYOCD and ACTA2.^9,11,12^ Besides these related functions emphasizing the possible role of SENCR lncRNA in CAD pathogenesis, the growing list of the lncRNAs contributes to CAD susceptibility encourage us to evaluate SENCR lncRNA. AK143260 is a novel identified lncRNA regulates the cardiac lineage commitment *in vitro.*^15^ cTN1 is a natural antisense transcript (NAT) with adult myocardium-specific expression and critical role in the regulation of cardiac muscle contraction.^16^ Antisense non-coding RNA in the INK4 locus (ANRIL) was first described as a candidate gene in the CAD-susceptibility region, 9p21.^17^ Several polymorphisms were recognized in this gene associating with myocardial infarction (MI), cancers, type 2 diabetes mellitus, and CAD.^18^ ANRIL is highly transcribed in SMCs, endothelial cells, and immune cells.^19^ Moreover, an elevated level of ANRIL expression was quantified in atherosclerosis plaques, and it is now recognized as a biomarker for CAD.^20^ Michalik et al described a function of MALAT1 decoy lncRNA in the regulation of endothelial cell function and vessel growth.^21^

Single nucleotide polymorphisms are the sequence variants with sometimes, deleterious effects on the expression and function of the genes and so, may involve in the pathogenesis of human diseases.^22,23^ The examples of such functional SNPs was reported for exon 2 and 3 of the SENCR gene.^9^ To the best of our knowledge, this the first study examined the association of the rs555172 SNP of the SENCR lncRNA with the susceptibility to CAD. rs555172 results from G-to-A substitution in the upstream region of the gene with minor allele frequency (MAF) of 0.48 for the ancestral G allele. Gene expression regulatory elements are mostly clustered in the upstream region of the gene. Nucleotide variations may alter the affinity of transcription factors binding to these regulatory elements and consequently, aberrant the gene expression.^24^ Regarding the rs555172 polymorphism, there is no information implicating the exact location and its functional effect on the gene to help us better explain the results of the present study.

In this case-control study, only low-risk CAD patients were enrolled. The information about the history of diseases like diabetes, hypertension, and hypercholesterolemia in control group gained based on the self-declaration. Therefore, information bias is an issue. Lack of considering and comparing the level of triglycerides between groups is the main limitation of the study and future research in this field should take care of this. The number of included cases of the STEM1 and NSTEM1 are unspecified in our study.

In conclusion, the results of the present study provide no data supporting the role of SENCR lncRNA in the pathogenesis of CAD in Iranian population.

## Ethical approval

This study was approved by the Ethics Committee of Islamic Azad University, Arsanjan Brabch.

## Competing interests

The authors declare there is no conflict of interest.
